# Biocatalytic conversion of lignin model oligomer using a laccase-mediator system†

**DOI:** 10.1039/d4gc01720j

**Published:** 2024-06-21

**Authors:** Christopher W. J. Murnaghan, William G. Forsythe, Jack H. Lafferty, Gary N. Sheldrake

**Affiliations:** a School of Chemistry and Chemical Engineering, David Keir Building Stranmillis Road Queen's University Belfast United Kingdom C.Murnaghan@qub.ac.uk

## Abstract

The use of the laccase enzyme from the fungus *Trametes versicolor*, coupled with the mediator 1-hydroxybenzotriazole (1-HBT) has been shown to be effective for the biocatalytic conversion of a hexameric lignin model compound containing three of the most common linkages found in native lignin. Cleavage of the model takes place over a 24 hours period predominantly at the β-O-4 ether linkage to give a previously known β-5 dimer intermediate which in turn was rapidly consumed to further degradation products. There is also mass spectrometric evidence of repolymerisation of the β-5 dimer and other degradation intermediates to form higher oligomers. Mechanistic pathways to account for the major catalytic processes are proposed.

## Abstract 2

Employment of laccase from Trametes versicolor effects considerable cleavage in β-O-4 linkage of lignin model hexamer.

## Graphical abstract

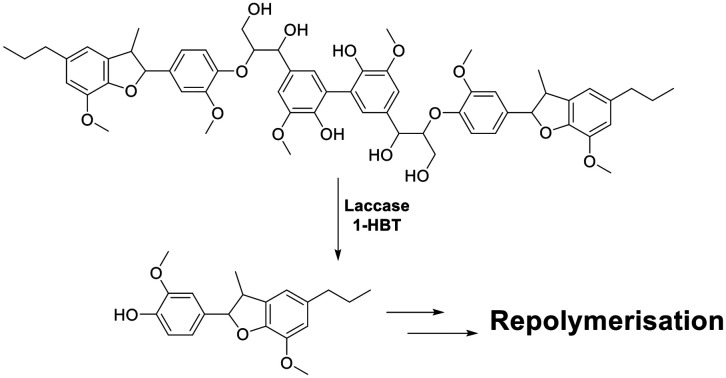

## Introduction

**Fig. 1:**
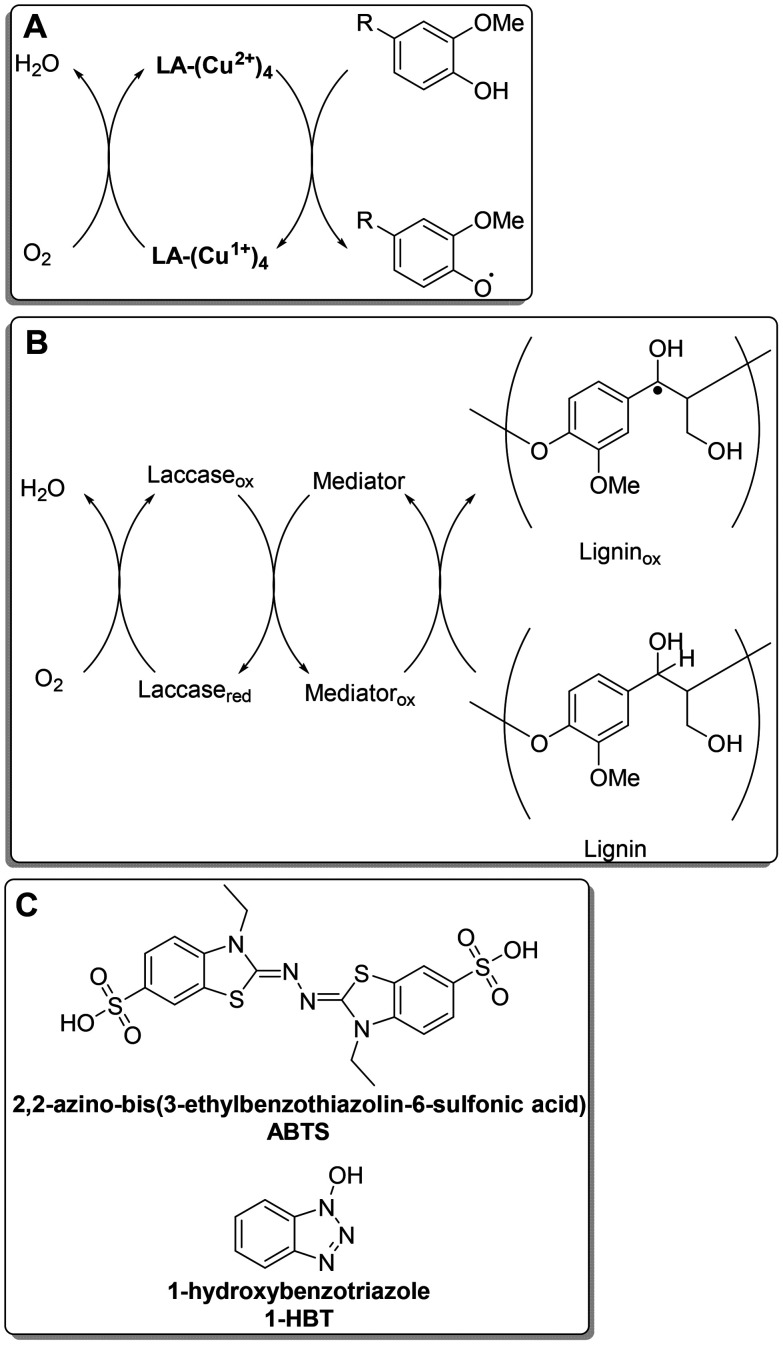
(A) Catalytic cycle of laccase producing phenoxyl radicals from phenolic lignin. (B) Catalytic cycle of laccase-mediator producing benzylic radical from non-phenolic lignin. (C) Commonly used mediators with laccase.

Crude oil is currently our main source of platform chemicals, of which around 50% are aromatic. Since crude oil is a finite resource, the bio-based production of platform chemicals is of increasing importance in research institutions and industry. The most well established of these bio-based platform chemicals is bio-ethanol and most other bio-based chemicals are aliphatic compounds.^1^ New sources of bio-renewable aromatic chemicals are therefore needed. Lignin is a complex polymer found in the cell walls of all higher plants^2^ and is the most abundant source of aromatic units in nature.^3^ Most isolated lignin (63 Mt per year worldwide) is an output of the Kraft and sulfite pulping processes used by the paper industry.^4^ Lignosulfonate depolymerisation is used on an industrial scale to produce vanillin but this is a rare example of the commercial production of an aromatic fine chemical from lignin.^5^ This, and other thermochemical processes used to depolymerise extracted lignin, require high temperatures and pressures, and most methods are unselective, producing a mixture of monoaromatics and unwanted side products.^3,6,7^ By potential means of circumventing these issues, interest in recent decades in using enzymes to catalyse reactions due to the low temperature and pressure and near-neutral pH of operation, high chemo-, regio- and stereoselectivities and biodegradability post-reaction.^8^ Four enzymes which degrade lignin have been isolated from white rot fungi.^9^ These are lignin peroxidase (LiP), manganese peroxidase (MnP), versatile peroxidase (VP) and laccase. The disadvantage of lignin peroxidase, manganese peroxidase and versatile peroxidase is that they are sensitive to even small excesses of hydrogen peroxide, the oxidising agent used by these enzymes, causing them to become permanently inactivated.^10,11^ By contrast, laccase uses oxygen as the oxidising agent^12,13^ and is sufficiently robust for use in industrial processes including bleaching denim, clarifying fruit juice and treating effluents.^14^ Laccase is a blue copper oxidoreductase, which oxidises four substrate molecules per catalytic cycle, releasing two molecules of water (Fig. 1A).^15^ Within their range of reactions, laccase enzymes oxidise phenols to phenoxyl including the free phenolic moieties within lignin. However, up to 85% of a lignin polymer is made up of non-free phenolic moieties,^16^ which have a redox potential of 1.06 V, so laccase alone is ineffective because it has a low redox potential of 470–790 mV, depending on the fungus species producing the laccase.^17,18^ However, laccase is able to oxidise mediators, which are small organic molecules which effectively shuttle electrons between lignin and resulting in an alternative mechanistic pathway through which the substrates can be consumed to enable oxidation of non-phenolic lignin residues^17,19–21^ (Fig. 1B). A commonly used mediator is 2,2-azino-bis-3-ethylbenzothiazoline-6-sulfonic acid (ABTS), which is recycled during the cycle,^20^ but some mediators (termed “enhancers”) are not reversibly oxidised, *e.g.* 1-hydroxybenzotriazole (Fig. 1C ^20^). Henceforth in this article, 1-HBT will be referred to as a mediator. Using laccase is advantageous because it selectively oxidises phenolic groups and benzylic C–H bonds along the lignin chain, and it operates at ambient temperature and pressure.^15^ A potential restriction is the need to use aqueous buffer, which is a poor solvent for many types of lignin; however, co-solvents can be added to improve lignin solubility.^22^ The extent of polymerisation is more significant when the lignin has a low molecular weight, contains sites available for cross-linking, and when the buffer or solvent dissolves or disaggregates the lignin and laccase is optimally active.^19,22–30^ In contrast, a laccase-mediator system usually favours depolymerisation, α,β-bond cleavage in the alkyl chain and α-(or benzylic) oxidation of lignin. Although a reduction in lignin molecular weight has been observed, the production of monoaromatics from industrially extracted lignin following laccase-mediator-catalysed depolymerisation has not yet been reported. This suggests that further optimisation of the reaction conditions is needed. Since waste or native lignins have complex structures, optimisation of laccase oxidation has been studied using simpler model compounds.^31,32^ Some lignin subunits can be cleaved into two monoaromatics (*e.g.* β-O-4′, β-5′),^33–36^ whereas other are only oxidised on their side chains and demethylated (*e.g.* 5-5′, lignostilbene- an ethene flanked by an aromatic ring on either side of the double bond)^36,37^ and are deemed recalcitrant and thereby preventing complete depolymerisation. A method for the optimisation of this process is the study of representative lignin model conversion biocatalytically.

**Fig. 2:**
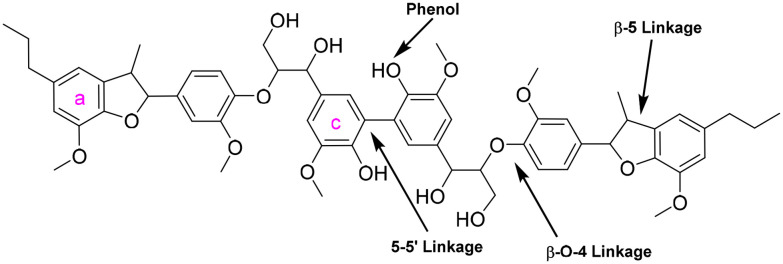
The structure of the lignin model hexamer. The bonds are labelled and are symmetrical about the centre of the molecule.

Sheldrake *et al.* have synthesized a lignin model hexamer (Fig. 2) containing three of the most common bonds in lignin, which makes it a complex, more representative model of lignin.^38^ The hexamer has a three-dimensional structure, by virtue of the aromatic rings being out of plane with each other. The mixture of stereoisomers in the hexamer is also representative of lignin.^39,40^ Despite the complexity of the structure, analysing the results of catalytic oxidations would be relatively simple because the material was uniform in length and also contains a mirror axis along the 5-5′ biphenyl linkage. The aim of this work was to study laccase-catalysed oxidation of the model hexamer to address a number of critical questions relating to lignin depolymerisation. Firstly, why the laccase/mediator-catalysed depolymerisation of authentic lignin does not proceed as far as producing monomers and what is preventing lignin from being depolymerised. Secondly, what effect the proximity of recalcitrant links (β-5′ and 5-5′) has on the cleavage of the more labile links (β-O-4′). Thirdly, whether it is repolymerisation or the recalcitrant 5-5′ biphenyl bond in lignin that prevent monoaromatics from being released. In order to efficiently depolymerise lignin for aromatic chemical production, an understanding of both depolymerisation and repolymerisation is required. The lignin model hexamer was found to be a good model of industrially-extracted lignin because, although depolymerisation was seen, no monoaromatics were produced and repolymerisation is also believed to have taken place. Laccase from *Trametes versicolor* (LTV) was chosen because it was commercially available with a high specific activity and high redox potential.^18,41^

## Results and discussion

### Optimisation of lignin model hexamer solubility *vs.* enzyme activity

**Fig. 3:**
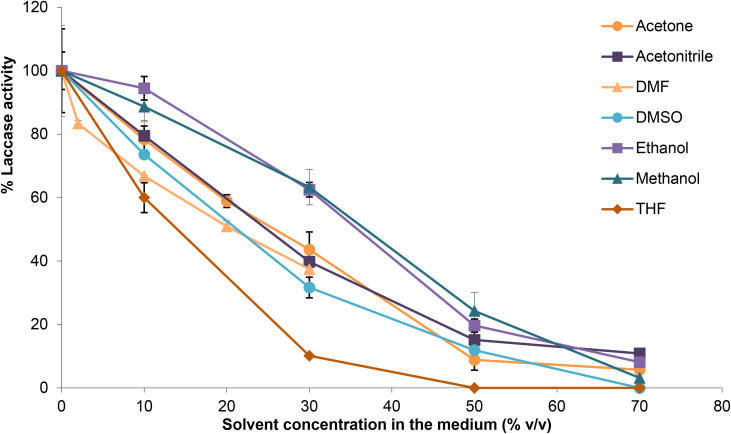
The effect of organic solvents on laccase activity.

In order for lignin to be effectively oxidised, it needs to be dissolved to be accessible to the laccase enzyme and the mediator and to avoid aggregation.^22,28,42,43^ Since the lignin model hexamer was not soluble in acetate buffer at pH 4.0–5.0 (the optimum pH range for LTV^14,44–47^) the first aim was to find a water-miscible organic solvent that could be mixed with the buffer to improve solubility of the hexamer while retaining laccase activity. A number of solvents have been tested previously for their compatibility with laccase, and the most enzyme-compatible solvents seem to be more hydrophilic and more polar,^22,48–50^ whereas generally for enzymes, non-polar solvents (having log *P* > 4) are required for high enzyme activity.^51^ Therefore all common, water-miscible organic solvents were tested (acetone, acetonitrile, DMF, DMSO, ethanol, methanol, THF). Catechol was chosen as the substrate for activity assays^52^ because it is soluble in both buffer and solvents and is relatively stable under these conditions.^49,53^ Laccase activity was tested in solvents mixed with water (Fig. 3). A reduction in efficiency of catechol oxidation was observed for all organic cosolvents tested but ethanol and methanol showed the lowest drop in activity of the enzyme. Moving forward, however, ethanol was proposed as the reaction co-solvent as it provided the greatest solubility of the hexamer. Hexamer solubility was tested by dissolving the hexamer at 3 mg mL^−1^ (3 mM) in each neat solvent and then diluting in water. The hexamer was soluble in all pure solvents but precipitated when buffer was added, with minimum solvent proportions required being 30% for ethanol, 40% for THF, 50% for acetonitrile, 60% for acetone, DMF and DMSO, and 70% for methanol. Ethanol, was therefore chosen as the solvent for oxidations of the lignin model hexamer because the minimum volume percentage at which the hexamer dissolved was 30% v/v, and this would minimise the concentration of solvent needed in the laccase reaction.

**Fig. 4:**
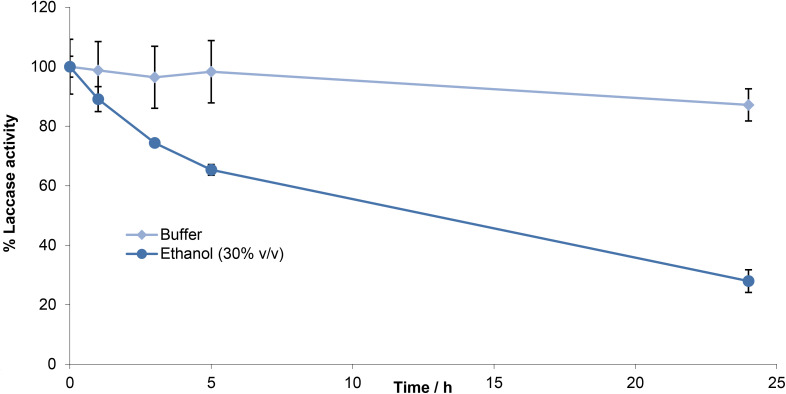
Residual activity of laccase after incubation in the presence of ethanol.

Since laccase-catalysed oxidation of lignin is relatively slow, the stability of the enzyme was assessed when incubated in the presence of 30% v/v ethanol at 30 °C. Although laccase lost 72% of its initial activity over 24 h (Fig. 4), there was still significant residual activity. Thus, any loss of activity would be compensated by the improved reactivity resulting from substrate solubility. The next step was to test for laccase-catalysed oxidation of the hexamer in this solvent system.

### Oxidation of the lignin model hexamer

1-Hydroxybenzotriazole (1-HBT) is a highly reactive mediator capable of inducing significant changes in lignin structure, lignin model dimers and DHP.^34,35,54,55^ Therefore, 1-HBT was chosen as the mediator for the laccase-catalysed oxidation of the hexamer. The lignin model hexamer was incubated with the laccase-mediator system in 30% ethanol/acetate buffer pH 4.0 at 30 °C, and sampled at intervals and analysed by HPLC. One of the major products formed, was identified as the β-5′ dimer (Fig. 7A), its place in the hexameric model can be seen in Fig. 2. Its identity was confirmed by both retention time and mass spectral matches with a synthetic β-5′ dimer standard, and its concentration was calculated using a standard calibration curve (ESI 2†). Similarly, in our other work involving this hexameric model compound,^56^ the β-5 dimer was released in a similar manner in the reaction. Clearly between the biocatalytic method described within and the previous photocatalytic study, there is a similarity when it comes to the reactivity of the hexamer particularly in the β-O-4 sidechain. However there was no repolymerisation of any fragments during the photocatalytic process which has been observed in this present study, repolymerisation which has been facilitated by the laccase/mediator system employed. This work presents evidence that there are significant bonding patterns which can be cleaved using biocatalytic means and similarly to the photocatalytic route, demonstrates that there is more than one method for the catalytic conversion of native lignin through lignin model compound studies.

**Fig. 5:**
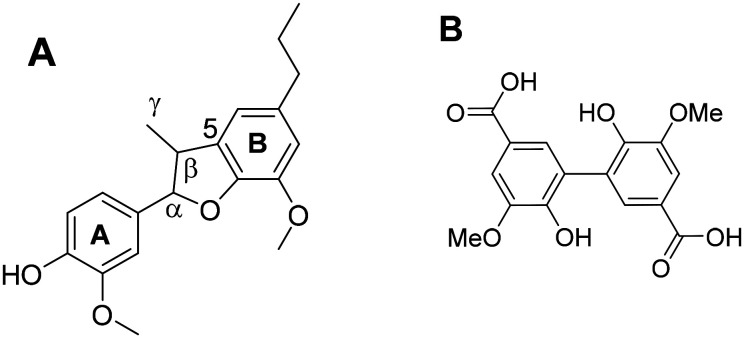
(A) The β-5′ dimer, a product of the laccase-catalysed oxidation of the hexamer. (B) Possible 5-5′ dimer by-product from the conversion of the hexamer.

The β-5′ dimer was formed by β-O-4′ bond cleavage in the hexamer. A possible mechanism, shown in ESI 7,† starts with hydrogen abstraction at the benzylic position.^3^ In theory, two moles of β-5′ dimer could be released per mole of the symmetrical hexamer. This could potentially leave an oxidised 5-5′ dimer (Fig. 5B) from the cleavage of both β-O-4′ bonds; however the 5-5′ dimer was not detected by HPLC, which could be because only one β-O-4′ bond was cleaved in each molecule, or because the 5-5′ dimer was rapidly consumed by the catalytic system.

The mechanism using laccase has precedence from Higuchi *et al.*^33^ and the mechanisms using a mediator are similar to those reported by Kawai *et al.*^55^ The higher abundance of β-5′ dimer in the presence of the mediator is more likely to be a result of the mediator being present in solution and facilitated by the loss of a benzylic proton; alternatively its abundance could be explained by a reduction in a competing path of polymerisation.

Within the first 30 minutes of shaking with the laccase/mediator it can be seen that there is a decrease in concentration of 0.22 mg mL^−1^. Considering the structure of the hexamer, there are several benzylic protons which can be abstracted to result in a radical cascade reaction such as the mechanism in ESI 7† resulting in consumption of the hexamer substrate.

**Fig. 6:**
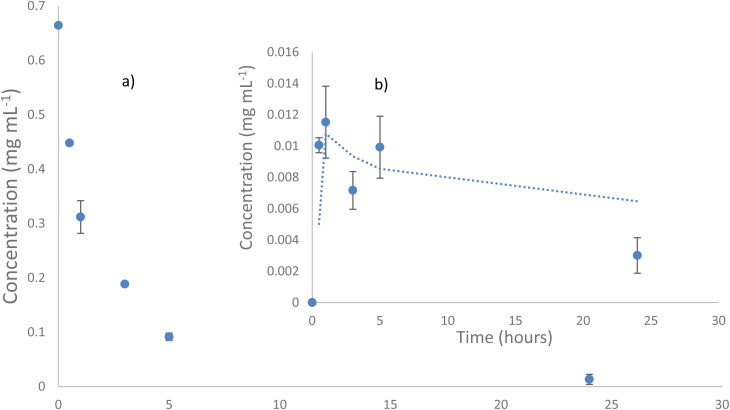
Concentration *vs.* time profile where (a) shows the removal of the hexamer lignin model compound with the laccase/1-HBT system within the 24 hours period and (b) the formation of the β-5 dimer which is present in the hexamer.

Fig. 6, insert (b) shows an interesting pattern of the β-5 dimer concentration within the reaction solution. There is an initial increase in the concentration as the dimer is cleaved from the hexamer with a maximum concentration being observed after 1 hour of reaction. There is then a decrease in the concentration of the dimer, most likely as a result of the dimer being consumed. The increasing and decreasing concentration of the liberated β-5 dimer from the hexamer as seen in Fig. 6(b) can be rationalised by the cleavage of the ether β-C–O bond to liberate the dimer and the subsequent consumption followed by cleavage at the ‘opposite end’ of the hexamer.

Considering also the mechanisms in ESI 7† the potential abstraction of a proton results in the formation of a phenoxide radical. Based on this proposed reactivity it is plausible for the β-5 dimer to react with another dimer molecule resulting in the formation of a tetramer.

### Ascertaining the repolymerisation of the β-5′ dimer

**Fig. 7:**
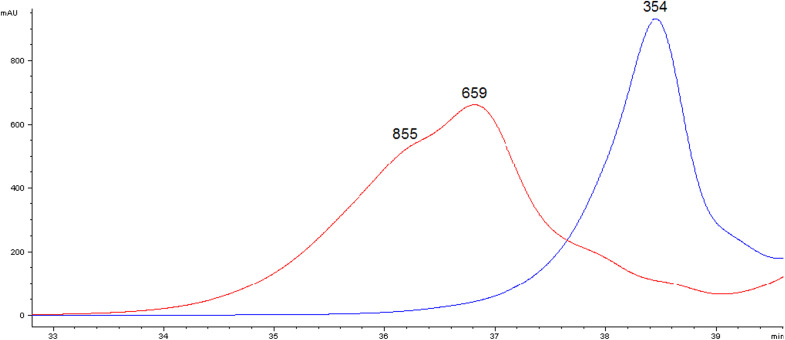
Effect of laccase/1-HBT-catalysed oxidation of the phenolic β-5′ dimer on the molecular weight distribution.

A remaining challenge was cleavage of the β-5′ bond to form monoaromatics. The β-5′ bond is important as it forms 9–12% of natural softwood lignin bonds.^57^ It is considered possible to cleave the β-5 bond, although slowly, as 99% of a 0.2 mM phenolic β-5 dimer solution was reported to be degraded in 48 h.^36^ Therefore, the aim was to investigate the oxidation of the β-5′ dimer by laccase/1-HBT under the same conditions as used in oxidising the hexamer, in order to know how the β-5′ dimer reacted once it was formed from the hexamer. These experiments were analysed using gel-permeation chromatography (GPC) and gas chromatography mass-spectrometry (GCMS) to determine and rationalise the formation of any products from the β-5 consumption. There were no easily-identifiable products in the GCMS chromatogram of the oxidation products, therefore GPC would be the more useful analytical tool for this work. Laccase polymerised the β-5′ dimer, as shown on the gel permeation chromatogram (to Mp 659 with a shoulder at Mp 855), even though it was in the presence of a mediator (Fig. 7). In the control the β-5′ dimer was untouched at Mp 354. The β-5′ dimer is a low molecular weight phenol and so could dimerise under laccase catalysis to form a tetramer, as a possible mechanism shows (ESI 8†). If one of these possible tetramers was acetobrominated, the molecular weight (*M*_w_ 697–739) would be roughly that of the product. GCMS showed 99 ± 2.0% of the β-5′ dimer was recovered from the controls, whereas 15 ± 0.25% was recovered in the 1 h samples, showing that laccase/1-HBT oxidised a significant proportion of the β-5′ dimer present (85%), which confirmed the GPC result. Leaving the reaction for an additional four hours did not significantly affect the amount of β-5′ dimer recovered (16 ± 0.51%) or the GPC profile, so the oxidation had terminated after 1 h. This work showed the difficulty of depolymerising a phenolic β-5′ dimer, as whilst the prior art showed it could be degraded, it is likely that it was turned into polymers.

To determine what the course of reaction was when the β-5 dimer was used as a substrate, LC-MS analysis of this reaction was employed. In the product stream, there were a number of interesting observations, it was seen that by matching retention time and also by found mass, vanillic acid was one of the major products formed during the laccase/mediator directed depolymerisation of the dimer.

The mechanism by which the vanillic acid is formed is largely not understood and as such proposals can only be made based on prior reported literature around the mechanisms associated with laccase/mediator systems. In terms of other LC-MS analyses of the reactions reported here, this is still ongoing work with a view towards having a full mechanistic analysis of the depolymerisation of the hexamer and determining the fate of the β-5 dimer which has been liberated from the hexamer in solution.

## Conclusions

The oxidation of a lignin model hexamer (Fig. 6a), which contained both phenolic and non-phenolic bonds, was investigated as a more realistic lignin model than the dimers used in the prior art, as it contained a variety of bonds and had a typical oligomer length. Oxidation of the hexamer was rapid, with ∼50% being oxidised in 1 h. The major product was a β-5′ dimer, which was expected as the 5-5′ bond is recalcitrant and the β-5′ bond is relatively difficult to cleave. Further work will need to find better conditions for cleavage of the β-5′ bond to form monoaromatics as the major products of β-5′ dimer oxidation were oligomers as evidenced by GPC analysis. Preferably this will occur in the same pot as rapid β-O-4′ bond cleavage.

The lignin model hexamer appears to be a good model of lignin, since it contains the recalcitrant bond types too, which slowed down the complete depolymerisation of the lignin model hexamer towards monoaromatics. Only the β-O-4′ bond could be rapidly cleaved. Unfortunately, the proximity of the labile β-O-4′ linkage did not lead to cleavage of the 5-5′ linkage, thus so far no cooperation in lignin depolymerisation has been seen.

Therefore, a hypothesis is that monoaromatics are not produced from lignin breakdown for two reasons: partly because the laccase/mediator system cannot break all the linkages and partly because those lignin fragments that contain the recalcitrant linkages polymerise when oxidised, *i.e.* they aid the polymerisation that laccase is already catalysing. This differs from the conclusion of Srebotnik and Hammel, who pointed out that the phenolic content of lignin allows rapid oxidation by laccase/1-HBT which leads to transient polymerisation, and that means lignin is not depolymerised efficiently. Their DHP was cleaved approximately two to three times and no further. In contrast, these results indicate that it is a combination of both recalcitrant linkages and repolymerisation that prevents lignin from being broken down entirely into its constituent monoaromatics. This hypothesis was reached because the structure of the lignin model was known, and one of the synthetic products was available, and so it was possible to identify that the recalcitrant linkages play an important double-edged role in preventing complete depolymerisation and monoaromatic release.

The β-O-4′ bond is reported to comprise 45–50% of linkages in natural lignin, however many of these bonds are broken during lignin isolation (the separation of lignin from cellulose) and replaced by condensed carbon–carbon bonds (5-5′, β-5′ and β-β′). Since the process of lignin depolymerisation to form aromatic chemicals should be rapid to be economically viable, it seems that β-O-4′ bonds should be retained in lignin, since these were the only ones which are proposed to have been cleaved in the lignin model hexamer and since the lack of β-O-4′ bonds neighbouring each other meant no monoaromatics were released. Therefore, new lignin isolation processes which are less damaging should be found to increase the proportion of labile linkages. In addition, knowledge of the way lignin structure effects enzymatic depolymerisation has generated thoughts about ‘designer lignin’, primarily the idea of introducing innovative chemically labile bonds into the lignin chain, including ester bonds and α-keto-β-ether linkages, as well as increasing the proportion of β-O-4′ linkages by increasing the amount of syringyl units produced by the plant. Other thoughts are to generate shorter lignin chains, more hydrophilic lignin chains, or lignin chains which cross-link to hemicellulose less often, all of which can be extracted from cellulose more easily. A final thought is to produce lignins which have valuable properties, such as linear lignins which could become carbon fibres or lignins with handles for functionalisation. This work adds to the argument for ‘designer lignin’. The laccase-catalysed depolymerisation of waste lignin (*e.g.* lignosulphonate, organosolv lignin and Kraft lignin), for the purpose of producing monoaromatic feedstock chemicals, is inefficient. In order to one day have a more efficient, economically-viable process for producing monoaromatics, this work employed a novel hexameric lignin model to better understand lignin depolymerisation.

## Data availability

The data supporting this article have been included as part of the ESI.†

## Conflicts of interest

There are no conflicts to declare.

## Supplementary Material

GC-026-D4GC01720J-s001
