# Correction: Biocatalytic conversion of lignin model oligomer using a laccase-mediator system

**DOI:** 10.1039/d5gc90198g

**Published:** 2025-11-04

**Authors:** Christopher W. J. Murnaghan, William G. Forsythe, Jack H. Lafferty, Alison Woodward, Gary N. Sheldrake

**Affiliations:** a School of Chemistry and Chemical Engineering, David Keir Building, Stranmillis Road, Queen's University Belfast UK C.Murnaghan@qub.ac.uk; b Faculty of Engineering, Coates Building, University Park, University of Nottingham UK

## Abstract

Correction for ‘Biocatalytic conversion of lignin model oligomer using a laccase-mediator system’ by Christopher W. J. Murnaghan *et al.*, *Green Chem.*, 2024, **26**, 10851–10858, https://doi.org/10.1039/D4GC01720J.

The authors regret the omission of one of the authors, Alison Woodward, from the original article. The corrected list of authors and affiliations for this paper is as shown above.

The Royal Society of Chemistry apologises for these errors and any consequent inconvenience to authors and readers.

