# Supplementary material for: Biocatalytic conversion of lignin model oligomer using a laccase-mediator system
Source: Green Chem. 2024 Jun 21;26(21):10851–8. doi: 10.1039/d4gc01720j (PMC11516409; doi:10.1039/d4gc01720j)
Supplement: GC-026-D4GC01720J-s001 [file GC-026-D4GC01720J-s001.pdf]

Electronic Supplementary Material (ESI) for Green Chemistry.
This journal is © The Royal Society of Chemistry 2024

# Biocatalytic Conversion of Lignin Model Oligomer Using a Laccase-Mediator System

Christopher W. J. Murnaghan[1]*, W. Graham Forsythe[1], Jack H. Lafferty[1], and Gary N. Sheldrake[1]

[1.] *School of Chemistry & Chemical Engineering, Queen's University Belfast, Belfast, BT9 5AG*

## Materials

Laccase from *Trametes versicolor* was purchased from BOC sciences with an activity of 13.6 U/mg using syringaldazine as the substrate. 1-Hydroxybenzotriazole hydrate (1-HBT), sodium acetate, acetic acid, acetyl bromide, and *N,O*-bis(trimethylsilyl)-trifluoroacetamide containing 10% trimethylchlorosilane were purchased from Sigma Aldrich. Ethanol, ethyl acetate, THF (HPLC grade, inhibitor free) and anhydrous magnesium sulfate were purchased from Fisher Scientific. Water was deionised by a Millipore system.

## Activity assay

Activity of laccase was measured in organic solvent/acetate buffer mixtures. Water-miscible organic solvent (up to 70% of the total volume), 0.1 M ammonium acetate buffer pH 5.0 and laccase (1.4 U/mL) were added to a quartz cuvette. After catechol (final concentration 250 mg/L, 2.3 mM) was added, the initial reaction rate was monitored at 405 nm (ε = 760 $M^{-1}$ $cm^{-1}$)at 30 °C [1]. Solvents were as shown in the legend, and rates are expressed as a percentage of the rate in buffer without solvent. Initial rates in 100% buffer were: Acetone (388 µM $min^{-1}$), acetonitrile and methanol (518 µM $min^{-1}$), dimethylformamide (DMF; 560 µM $min^{-1}$), dimethylsulfoxide (DMSO; 491 µM $min^{-1}$), ethanol and tetrahydrofuran (THF; 429 µM $min^{-1}$). Error bars show standard deviations for duplicate assays.

## Enzymatic reaction

## Oxidation of the lignin model hexamer

A reaction mixture (500 mL in a 2 L Erlenmeyer flask) contained hexamer (0.25 mM), 1-HBT (3 mM), and laccase (6 U/mL) in ethanol/acetate buffer (0.2 M, pH 4.0) 30/70 v/v, and was incubated at 30 °C with 200 rpm shaking for 24 h. At 0, 0.5, 1, 3, 5 and 24 h, a sample (80 mL) was taken, extracted into ethyl acetate, dried in vacuo. Following the concentration in vacuo the residue was taken up in 1.5 mL of methanol and samples run on HPLC–UV, an Agilent 1100 HPLC system equipped with a photodiode array UV-Vis was used. A Phenomenex KinetexR PhenylHexyl analytical column (150 mm ×4.6 mm, 100 Å) was used, and the mobile phase was (A) 0.2% acetic acid and (B) methanol over the following gradient: 50:50 (A:B) to 100% B (0-6 min) and then back to 50:50 (A:B) (6-10 min). The flow rate was 1 mL $min^{-1}$ and the injection volume was 1 µL. The hexamer standard eluted at 7.083 minutes. The error bars are calculated from the replicates of the reaction.

**Ascertaining the repolymerisation of the β-5' dimer**

A reaction mixture (167 ml in a 500 ml Erlenmeyer flask) contained β-5' dimer (0.5 mM), 1-HBT (3 mM), and laccase (6 U/ml) in ethanol/acetate buffer (0.2 M, pH 4.0) 30/70 v/v, and was incubated at 30 °C with 200 rpm shaking for 5 h. A control omitted laccase. At 1 and 5 h, a sample (80 ml) was taken, extracted into ethyl acetate, dried in vacuo, and split 50:50 for GCMS and GPC analysis. GPC samples were acetobrominated and dissolved in THF before analysis.

**Sample preparation**

The sample was partitioned between ethyl acetate and buffer (5:3). The organic layer was washed with saturated NaCl solution, dried over anhydrous $MgSO_4$ and evaporated under reduced pressure.

Where a sample was taken for both GPC and GCMS, the starting material and products were re-dissolved in 10 ml ethyl acetate with the aid of sonication, and split 50:50 between two 10 ml round bottom flasks, then dried. One flask was intended for GCMS and the other was intended for GPC.

**Characterisation of the volatile aromatic degradation products by GC-MS**

The residue was trimethylsilylated [*N,O*-bis(trimethylsilyl)-trifluoroacetamide containing 10% trimethylchlorosilane, and pyridine (5/3, v/v)], volume 0.8 ml. Authentic standards were trimethylsilylated and calibration curves created in order to confirm concentration of products and identity products by retention time and mass spectrum. The pyridine solutions were analysed using an Agilent 7890A GC-MS system equipped with a Restek Rxi-5ms capillary column (0.25 mm by 30 m by 0.25 µm) and a 5975C inert mass selective detector (MSD) with a quadrupole mass analyser. Mass spectra were acquired using positive ion electron impact ionization at 70 eV with the source temperature at 300 °C. Helium was used as a carrier gas at 0.55 ml/min. 1 µl was injected at an inlet temperature of 250°C and at a split ratio of 65:1. The oven temperature was kept at 70°C for 5 min, increased to 300°C at 7°C/min, and then held for 5 min. Data analysis was done using the Agilent MSD ChemStation software.

**Molecular weight distribution of products by GPC**

The extract was dissolved in acetic acid (2.3 ml, 30 min) and derivatised using acetyl bromide (0.25 ml, 60 min) at room temperature. The acetic acid was evaporated under reduced pressure and the dry residue redissolved in 10 ml THF. The starting material was derivatised likewise and redissolved in 1 ml THF per mg of material weighed out. 30 µl samples were analysed by GPC using two tandem Styragel columns (HR-5E and HR-1, Waters). The columns were eluted with a flow rate of 0.5 ml/min at room temperature for 60 min with THF. The products were monitored by UV spectroscopy at 280 nm, with the flow also passing through a refractive index detector. A calibration curve of ReadyCal polystyrene standards (Waters; SI 6) allowed the molecular weights of acetobrominated products to be estimated.

**Synthetic schemes and experimental detail**

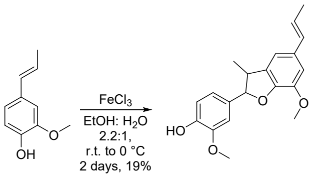


Isoeugenol (5.0 g, 30 mmol) was dissolved in water and EtOH (25 mL and 57 mL, respectively). To this was added $FeCl_3$(2.96 g, 18.2 mmol, 0.6 eq). The flask was stoppered and shaken vigorously for 30 s and then stored in the freezer for 3 d. The mixture was filtered under vacuum and the solid washed with ice-cold EtOH. The crystalline product was then resuspended in a minimum quantity of DCM and passed through a short silica plug, where then the plug was washed with DCM (100 mL) and the filtrate concentrated to provide the product as a white solid (0.94 g, 19% yield).

$^{1}$H NMR (400 MHz, $CDCl_3$) δ ppm 1.38 (3 H, d, J=6.77 Hz, γ′-H), 1.87 (3 H, d, J=6.53 Hz, γ′′-H), 3.45( 1H, m, β′-H), 3.88 (3 H, s, OMe), 3.89 (3 H, s, OMe), 5.10 (1H, d, J=9.45Hz, α′-H), 5.62 (1H, s, OH), 6.12 (1H, m, J=15.68 Hz, 6.59 Hz, Ar), 6.90 (2H, s, Ar), 6.97 (1 H, s, Ar).

$^{13}$C NMR (400 MHz, $CDCl_3$) δ ppm 17.57, 18.40, 45.64, 55.94, 55.99, 93.92, 108.93,109.23, 113.32, 114.08, 120.00, 123.52, 130.94, 132.1, 132.22, 133.28, 144.17, 145.79, 146.59, and 146.68.

Values consistent with reported literature[3,4]

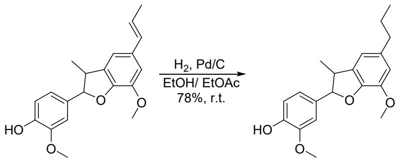


β-5 (1.09 g, 3.34 mmol) added to flask and made solution by addition of EtOH and EtOAc (20 mL each respectively to total 40 mL) and the flask was then purged with nitrogen three times before addition of Pd/C (10% loading, 0.109 g). Following this, the flask was evacuated three times under vacuum and then purged ten times with hydrogen by balloon. The reaction was allowed to stir at room temperature for 3-4 hours before the reaction solution was passed through a pad of celite and the filtrate stripped down to provide a brown/ black oil. Oil was chromatographed on silica gel with elution gradient 7:1 to 5:1 P.E: EtOAc to provide the product as an off-white solid.

$^{1}$H NMR (400 MHz, $CDCl_3$) δ ppm 0.96 (t, 3H,, J= 7.40 Hz, γ′′-$CH_3$), 1.37 (d, 3H, J= 6.86 Hz, γ′-$CH_3$), 1.64 (m, 2H, β′′-$CH_2$), 2.55 (t, 2H, J= 7.58 Hz, α′′-$CH_2$), 3.44 (m, 1H, β′-H), 3.88 (os, 6H, 2x OMe), 5.08 (d, 1H, J= 9.53 Hz, α′-H), 5.61 (s, 1H, ArOH), 6.60 (d, 2H, J= 13.61 Hz, Ar), 6.90 (m, 2H, Ar), 7.00 (d, 1H, J= 1.67 Hz, Ar)

$^{13}$C NMR (300 MHz, $CDCl_3$) $\delta_H$ ppm 13.90, 17.40, 25.07, 38.09, 45.80, 55.99, 56.02, 93.62, 108.99, 111.83, 114.04, 115.44, 119.99, 131.94, 132.97, 136.32, 143.87, 145.39, 145.73, 146.66

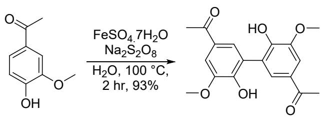


Acetovanillone (10 g, 60.2 mmol) was added to 1 L flask and made into solution by addition of boiling water (300 mL), when complete dissolution was achieved iron sulphate heptahydrate (0.837 g, 3.01 mmol, 0.05 eq) followed immediately by sodium persulphate (7.19 g, 30.2 mmol, 0.5 eq) which turned the solution from deep green to produce a brown

precipitate. Precipitate filtered under vacuum and then suspended in boiling water, to boiling water suspension was added solid sodium hydroxide until precipitate dissolved, final precipitation was performed by adding a few drops of concentrated hydrochloric acid where solid was then filtered under reduced pressure and then stored in oven overnight to dry solid of water.

$^{1}H$ NMR (400 MHz, $CDCl_3$) δ ppm 2.58(6H, s, COMe), 4.02(6H, s, OMe), 6.32(2H, s, OH), 7.61(4H, d, J=10.01Hz, Ar)

$^{13}C$ NMR (400 MHz, $CDCl_3$) δ ppm unsuccessful due to insolubility issues in all available deuterated solvents

$^{1}H$ NMR shifts consistent with reported literature [5]

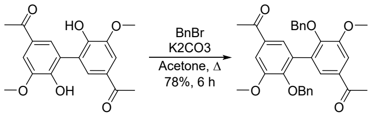


Dedihydroacetovanillone (5g, 15.1 mmol) dissolved in acetone 50mL and brought to reflux, $K_2CO_3$ (4.17g, 30.2 mmol, 2 eq) added followed by BnBr (3.6 mL, 30.3 mmol, 1 eq) allowed to reflux overnight. Reaction mixture cooled and the filtered, filtrate concentrated to black oil, oil dissolved in EtOAc and passed through large silica plug, filtrate then concentrated to orange oil which crystallised on standing.

$^{1}H$ NMR (300 MHz $CDCl_3$) $\delta_H$ ppm 2.47(6H, s, COMe), 3.97(6H, s, OMe), 4.89(2H, s, $CH_2Ph$), 6.99-7.03(4H, m, Ar), 7.14-7.16(6H, m, Ar), 7.42(2H, s, Ar), 7.61(2H, s, Ar)

$^{13}C$ NMR (300 MHz $CDCl_3$) $\delta_H$ ppm 26.24, 55.98, 74.51, 110.72, 125.11, 128.67, 128.94, 131.87, 132.54, 137.06, 149.84, 152.95, 196.81

MS calculated 510.59, mass found [M+ $H^+$] = 511.21

Values obtained are consistent with reported literature [4]

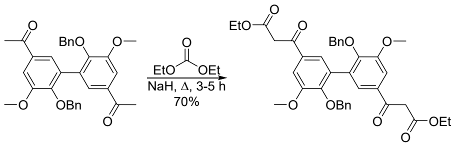


Dibenzylated species (10g, 19.6 mmol) was dissolved in diethyl carbonate (22.85mL, 188.6 mmol, 9.6 eq) and heated to 80°C, to this solution was added sodium hydride (60% dispersion in oil, 3.9g, 97.5 mmol, 4.97 eq) with caution, reaction monitored by NMR spectroscopy. When finished, the reaction mixture was cooled and carefully quenched with water (10mL), then diluted with EtOAc (50mL) and diluted further by the addition of water (40mL). Organic product extracted and washed with water, organic product concentrated to produce dark brown oil, trituration with hexane or petroleum ether was used to remove excess diethyl carbonate. Flash column chromatography with elution gradient 5:1 to 3:1 P.E: EtOAc, was used to purify further the product, providing white solid.

$^{1}H$ NMR (300 MHz, $CDCl_3$) $\delta_H$ ppm 1.26 (6H, t, 7.04 Hz, $CH_3$), 3.82, (4H, s, $CH_2COOEt$), 3.98 (6H, s, OMe), 4.18 (4H, q, 7.14 Hz, $CH_2CH_3$), 4.88 (4H, s, $OCH_2Ar$), 6.98-7.00 (4H, m, Ar), 7.15-7.18 (6H, m, Ar), 7.29 (2H, s, Ar), 7.61 (2H, s, Ar)

$^{13}C$ NMR (300 MHz, $CDCl_3$) $\delta_H$ ppm 14.12, 45.59, 65.13, 61.43, 74.66, 111.23, 125.03, 127.92, 128.1, 128.14, 131.42, 131.94, 136.95, 150.45, 153.14, 167.53, 191.50

MS calculated 654.71, mass found [M+ $Na^+$] = 677.24

Values obtained are consistent with reported literature [4]

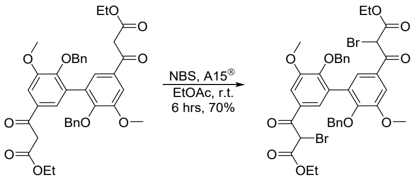

Flask charged with bis-β-keto ester (8.11 g, 12.4 mmol) and made into solution by addition of EtOAc (200 mL), to yellowish solution was then added N-bromosuccinimide (4.34 g, 24.4 mmol, 2 eq) followed by Amberlyst A15 (1.6 g). Yellow solution then allowed to stir at room temperature for 6 hours or until complete consumption of starting material was observed by TLC with eluent 2:1 P.E: EtOAc anisaldehyde stain. When reaction was complete, solid acid catalyst was filtered off and the reaction mixture stripped down to provide orange oil, this was chromatographed on silica gel to provide the product as a yellow oil in 70 % yield.

$^{1}$H NMR (300 MHz, $CDCl_3$) $\delta_H$ ppm 1.23 (6H, t, 7.04 Hz, $CH_3$), 3.98 (6H, s, OMe), 4.25 (4H, q, 7.14 Hz, $CH_2CH_3$), 4.91 (4H, s, $OCH_2Ar$), 5.53 (2H, s, CHBr), 6.99-7.02 (4H, m, Ar), 7.14-7.20 (6H, m, Ar), 7.39-7.40 (2H, s, Ar), 7.64 (2H, s, Ar)

$^{13}$C NMR (300 MHz, $CDCl_3$) $\delta_H$ ppm 13.94, 14.21, 21.06, 45.87, 56.17, 60.41, 63.30, 74.76, 112.31, 125.38, 127.95, 128.08, 128.17, 128.59, 131.89, 136.83, 150.98, 153.19, 165.13, 171.19, 187.12

MS calculated 812.50, mass found [M+H$^+$] = 830.72

Values obtained are consistent with reported literature [4]

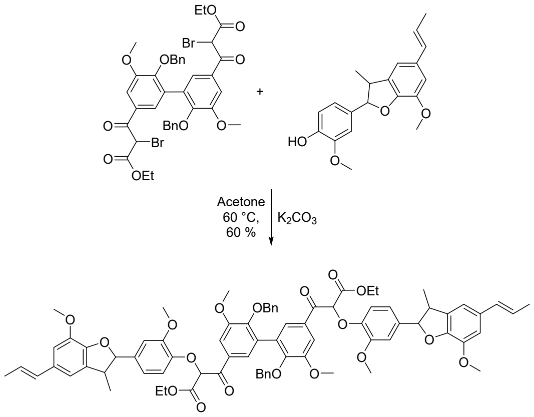

Flask charged with dehydrodiisoeugenol (3.38 g, 0.01 mol) and made into solution by addition of acetone (30 mL), to colourless solution was then added $K_2CO_3$ (1.38 g, 0.01 mol, 1eq). Brominated intermediate (4.21 g, 0.005 mol, 0.5 eq) dissolved in acetone (20 mL) and added in one portion to dehydrodiisoeugenol solution. Reaction allowed to stir at 60 °C and monitored by TLC with eluent 3:1 P.E: EtOAc anisaldehyde stain. When consumption of dibromo intermediate was observed by TLC the reaction mixture was filtered under gravity and filtrate stripped down to produce dark oil which was chromaographed on silica gel with elution gradient 6:1 to 4:1 P.E: EtOAc to provide the product as an off-white solid in 60% yield.

$^{1}$H NMR (400 MHz, $CDCl_3$) δH (ppm): 1.17 (6H, t, J = 7.76 Hz, $COOCH_2CH_3$), 1.34 (6H, d, J = 7.52 Hz, γ′-H), 1.86 (6H, two doublets superimposed, J= 6.76 Hz γ′′-H), 3.37 (2H, m, β′-H), 3.62-3.94 (multiple singlets corresponding to ArOMe), 4.18 (4H, q, J= 7.00 Hz, $COOCH_2CH_3$), 4.88 (4H, overlapping singlets, $OCH_2Ar$), 5.06 (2H, 3 sets of overlapping doublets, J= 9.04 Hz, α′-H), 5.75 (2H, two overlapping singlets, β-H), 6.09 (2H, dq, J= 6.9, 9.00 Hz, β′′-H), 6.35 (2H, d, J= 6.35 Hz, α′′-H), 6.73-7.11 (20H, m, Ar), 7.70 (2H, m, Ar), 7.79 (2H, m, Ar)

$^{13}$C NMR (100 MHz, $CDCl_3$) δC ppm- 14.16, 17.92, 18.51, 45.75, 45.78, 55.83, 56.07, 56.20, 62.25, 74.71, 82.47, 93.34, 109.40, 110.63, 110.73, 113.16, 113.46, 119.03, 119.14, 123.69,

125.97, 127.83, 127.92, 127.99, 128.03, 128.16, 129.63, 131.04, 132.45, 132.59, 132.62, 133.22, 136.43, 137.30, 144.28, 146.15, 146.62, 150.72, 150.79, 151.13, 152.89, 167.01, 190.32

MS calculated 1303.46, mass found [M+$NH_4^+$] = 1321.73

Values obtained are consistent with reported literature [4]

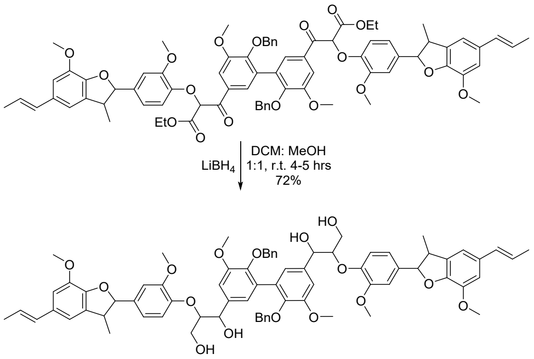


Flask charged with ketoester (0.5 g, 3.84 mmol) and made into solution by addition of methanol (10 mL) and DCM (10 mL), when complete dissolution was observed, the solution was cooled to 0 °C. Lithium borohydride (8.35 mg, 3.84 mmol, 1 eq) was added in one portion and the reaction monitored by TLC with eluent 10:1 DCM: MeOH anisaldehyde stain. When complete consumption of starting material was seen the reaction was quenched by addition of $H_2O$ (15 mL), crude product was extracted with ethyl acetate and washed thoroughly with water and brine (2x 20 mL each). Crude organic dried over $MgSO_4$, filtered and concentrated under reduced pressure to provide the crude as a colourless oil which was chromatographed on silica gel with elution gradient 30:1 DCM: MeOH isocratic solution to provide the product in 72 % yield.

$^1$H NMR (400 MHz, $CDCl_3$) δH ppm 0.88 (d, J= 7.2 Hz, γ′-H-cis)1.38 (6H, two overlapping doublets, J= 6.80 Hz, γ′-H ) , 1.86 (6H, two overlapping doublets, J= 6.52 Hz, γ′′-H), 3.43 (m, 2H, J= 7.04 Hz,β′-H) , 3.49-3.79 (region is shown to be overlapping γ-H from a mixture of erythro and threodiastereoisomers as well as containing β′-H-cishydrogen) , 3.82-3.88(multiple singlets corresponding to ArOMe), 3.98 (m, β-H from Threo diastereoisomer), 4.12(broad m, A ring β-H from Erythrodiastereoisomer), 4.76 (4H, overlapping signals, CH2Ar), 4.95 (2H, overlapping doublets, J= 5.10 Hz, α′-H), 5.10 (2H, overlapping signals, β-H),5.74 (d, J=7.2 Hz, α′-H cis), 6.11(2H, overlapping doublet of quintets, J= 6.52, 15.56 Hz, β′′-H), 6.35 (2H, overlapping doublets, J= 15.56 Hz, α′′-H), 6.75-7.14 ( m, Ar)

$^{13}$C NMR (100 MHz, $CDCl_3$) δC ppm- 17.88, 18.44, 45.71, 45.75, 56.01, 56.05, 56.13, 60.77, 72.90, 72.98, 74.59, 87.03, 93.24, 93.33, 109.37, 109.40, 109.71, 110.22, 110.27, 110.33, 113.42, 119.85, 119.90, 120.49, 121.25, 123.64, 127.53, 127.75, 127.85, 127.94, 128.06, 130.97, 132.44, 132.73, 133.16, 136.24, 137.89, 144.21, 145.13, 146.53, 146.88, 151.57, 151.64, 153.16

MS calculated 1223.42, mass found [M+$NH_4^+$] = 1241.68

Values obtained are consistent with reported literature [4]

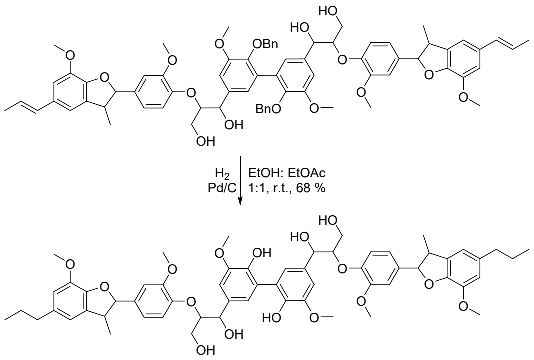


Flask charged with polyol (200 mg, 1.63 mmol) and made into solution by addition of EtOH (10 mL) and EtOAc (10 mL), flask purged with $N_2$, and 10% Pd/C (20 mg) was added to solution. Flask was then purged with $H_2$ eight times and allowed to stir at room temperature until complete consumption of the starting material was observed by TLC with elution gradient 5:1 DCM: MeOH anisaldehyde stain. When complete consumption was observed, reaction solution was passed through short celite pad under light vacuum, filtrate stripped down to provide dark oil which was chromatographed on silica gel with elution gradient 25:1 to 20:1 DCM: MeOH to provide the product in 68 % yield.

$^1$H NMR (400 MHz, $CDCl_3$): δ 0.79 (d, J= 7.3 Hz, γ′-H-cis), 0.96 (6H, t, J=7.28 Hz, γ′′-H), 1.38 (6H, two doublets superimposed, J=6.52, 6.76 Hz, γ′-H), 1.64 (4H, pseudo-quintet, J=7.80, 7.52, 7.28 Hz, β′′-H), 2.55 (4H, pseudo-triplet, J= 7.28, 8.04 Hz, α′′-H), 3.44 (2H, m, β′-H), 3.55 (superimposed, β′-H-cis), 3.55-3.95 (m, γ-H), 3.84-3.89 (multiple singlets corresponding to ArOMe and overlapping γ-H from a mixture of erythro and threo 3 diastereoisomers as well as containing β′-H-cis hydrogen), 4.05 (m, β-H from threo diastereoisomer), 4.18 (broad m, β -H from erythro diastereoisomer), 4.98 (broad m, 2H, α-H), 5.09 (2H, two doublets superimposed, J=9.52 and 9.28 Hz, α′-H), 6.15 (four singlets, ArOH), 6.60 (d, 4H, Ar, J=14.28 Hz ), 6.88-7.10 (m, 10H, Ar)

$^{13}$C NMR (100 MHz, $CDCl_3$) δC ppm- 13.93, 17.60, 25.10, 29.27, 38.09, 45.82, 55.97, 55.99, 56.21, 60.95, 72.87, 87.17, 92.96, 108.45, 110.33, 111.86, 115.43, 120.03,120.51, 121.22, 122.24, 124.12, 131.78, 132.97, 136.55, 142.16, 142.60, 143.87, 145.23, 146.81, 146.89, 147.44, 151.26, 151.53

MS calculated 1047.20, mass found [M+$NH_4^+$] = 1064.50

Values obtained are consistent with reported literature [4]

## Supplementary information

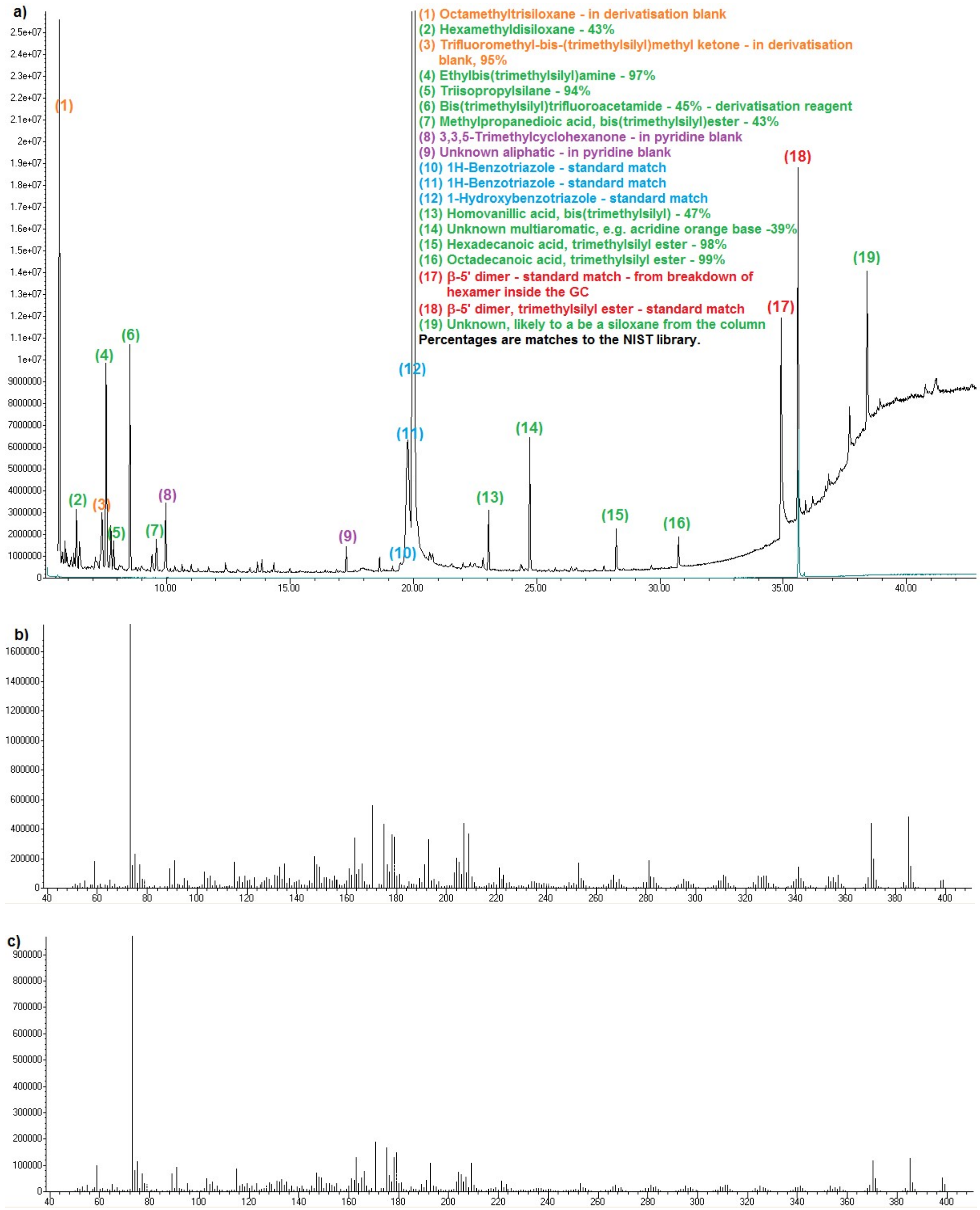


**SI 1: No monoaromatics were detected in the GCMS chromatograms, so the β-5' dimer was the only lower molecular weight product formed by depolymerisation of the lignin model hexamer, as this 3 h chromatogram showed next to the 4.3 mM synthetic standard (a). This example was typical of samples at other time points. The mass spectrum of the trimethylsilylated synthetic β-5' dimer (c) also matched the product's mass spectrum (b).**

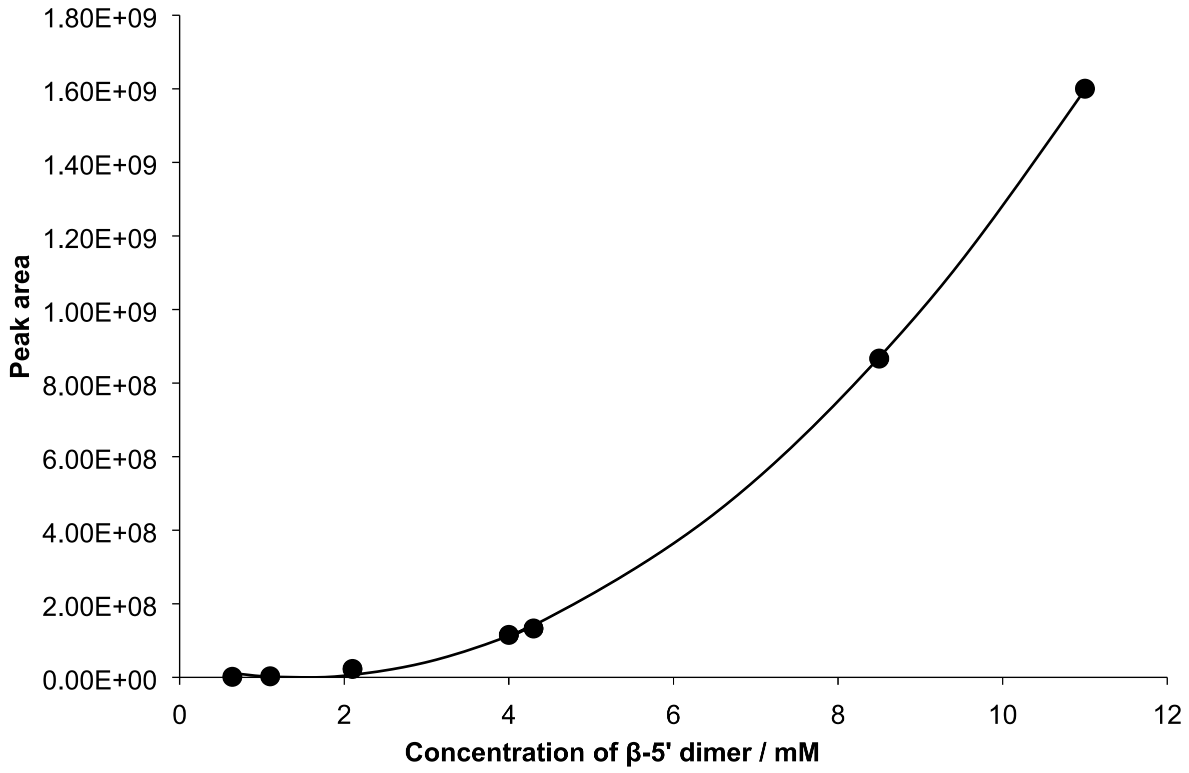


**SI 2: Calibration curve of β-5′ dimer standards (GCMS)**

Lignin model hexamer starting material (11.4 mg) was weighed out and derivatised for GCMS using the same volumes of reagents as the samples used. The peak area of β-5′ dimer (67160780) was converted into the concentration in the derivatisation mixture (3.41 mM) using the calibration curve of standards. That was converted into the concentration in a typical sample's derivatisation mixture (2.99 mM) by multiplying by the ratio between the mass of hexamer in a sample and the mass weighed out (10 mg/11.4 mg). That was converted to the concentration in a typical 40 ml sample (0.0599 mM) by multiplying by the volume ratio (0.8 ml/40 ml). The % yield was calculated (12%) with the theoretical yield being 0.5 mM.

**SI 3: Calculation of the concentration of β-5′ dimer that would be in a solution of the unreacted starting material standard. The same procedure was used for samples except that it started with the residue from the ethyl acetate extraction and there was no need to multiply by a mass ratio.**

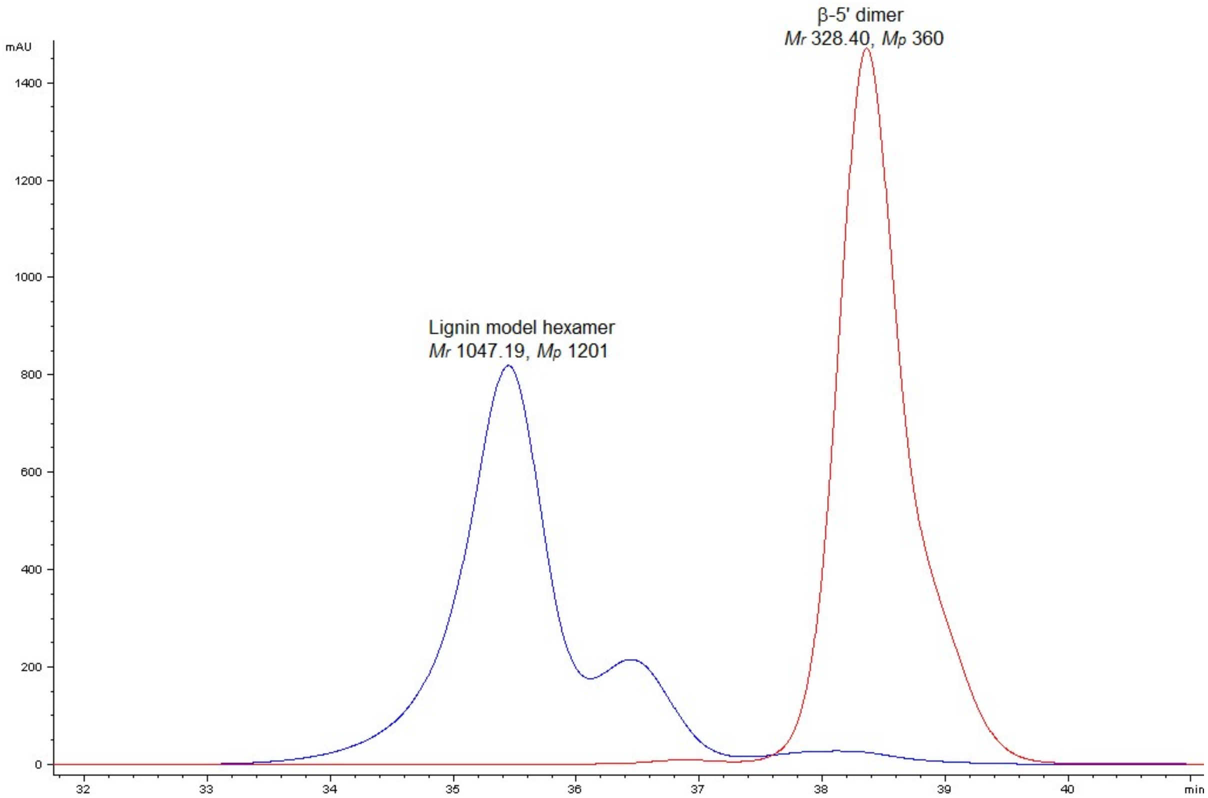


**SI 4: Acetobrominated standards of lignin model hexamer and synthetic β-5' dimer (1 mg/ml)**

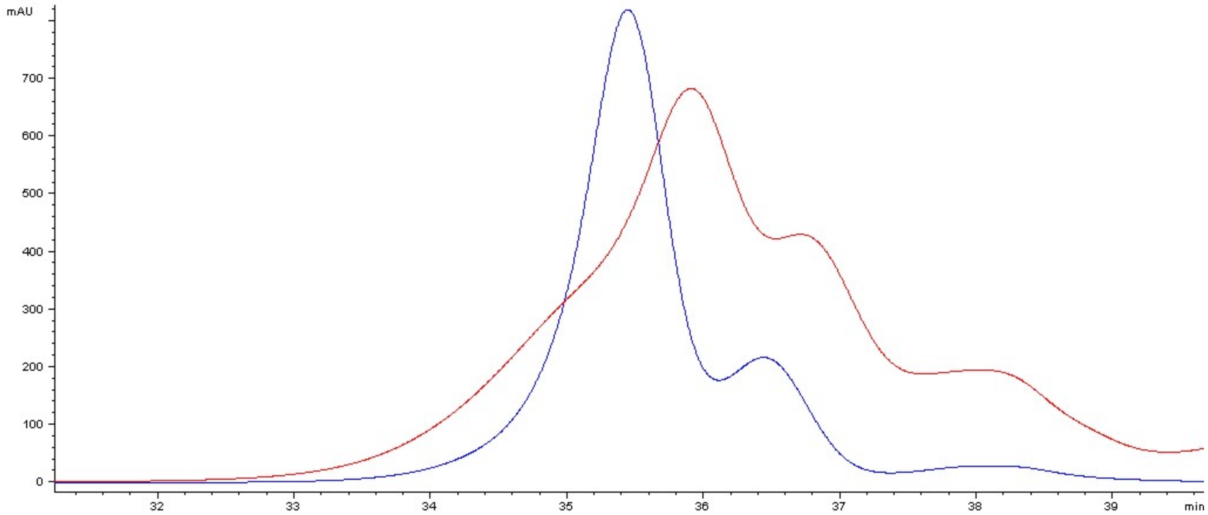


**SI 5: Overlaid chromatograms of the standard lignin model hexamer (1 mg/ml) and the sample of hexamer after 1 h oxidation by laccase/1-HBT system. Comparing the peak heights showed that the concentration of hexamer in THF at 1 h was ~ 1 mg/ml, and since the starting concentration of hexamer in THF would have been 2.1 mg/ml (since the reaction concentration was 0.26 mg/ml and it was concentrated 8 times), ~ 50% of the starting material must have reacted in 1 h.**

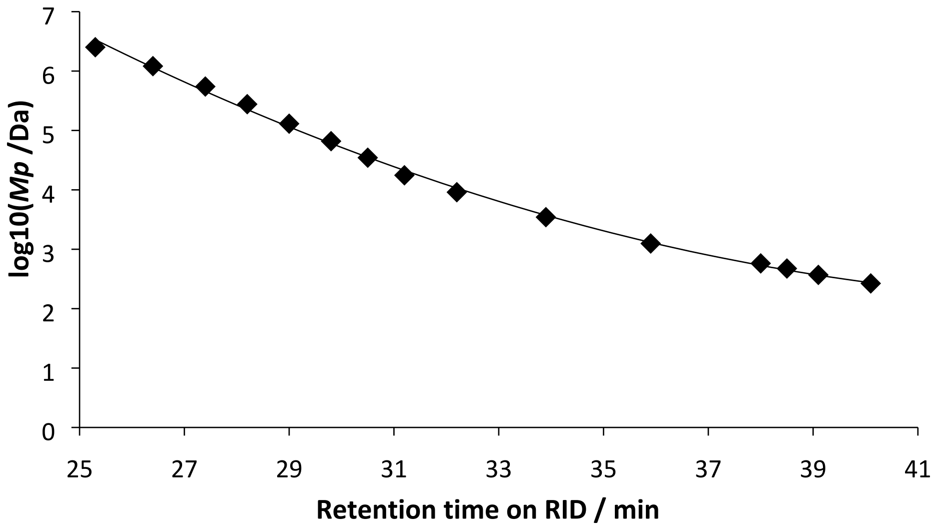


**SI 6: Calibration curve of Waters ReadyCal polystyrene standards (GPC)**

## Proposed Mechanisms

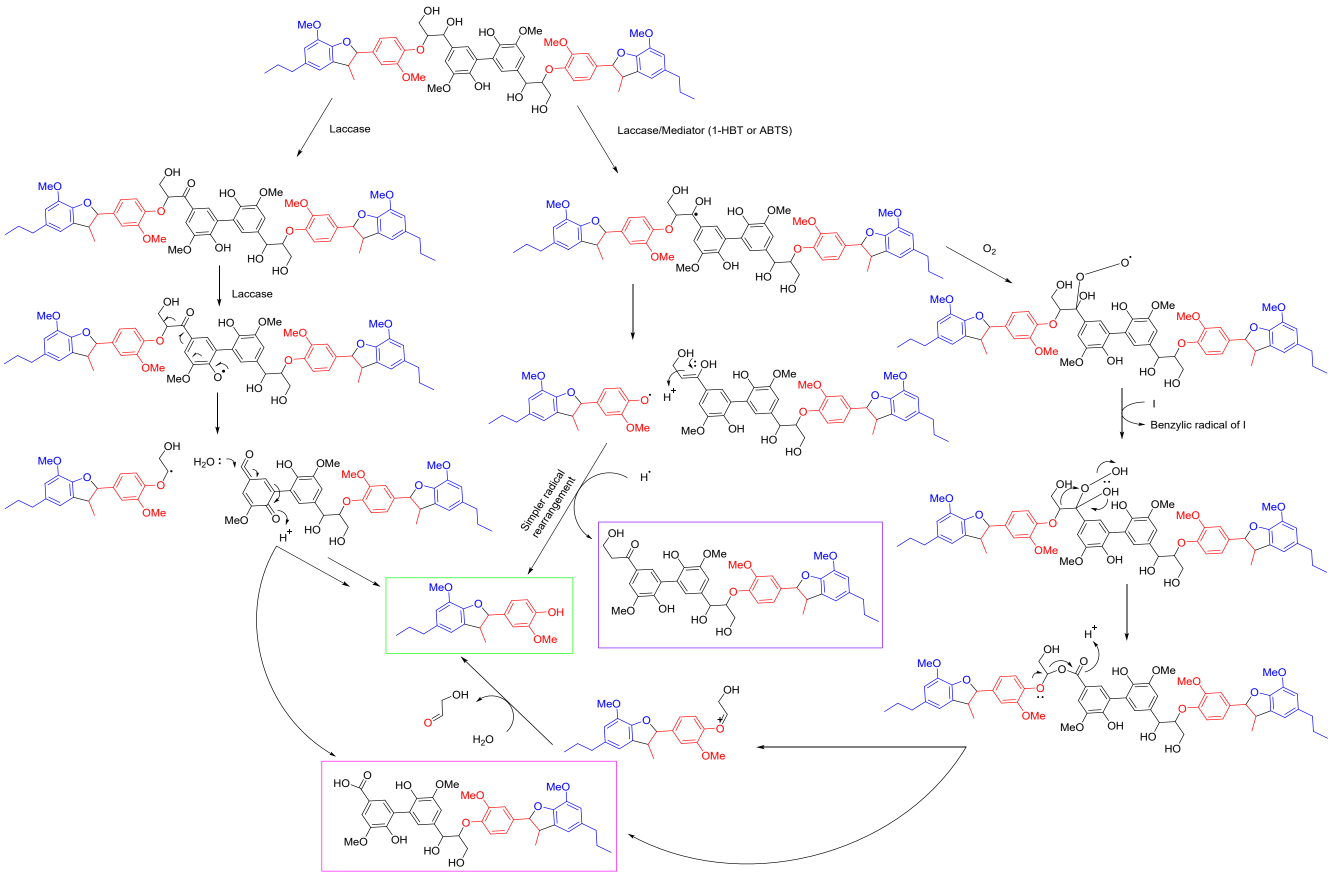


**SI 7: Possible pathways of β-5’ dimer formation from the hexamer**

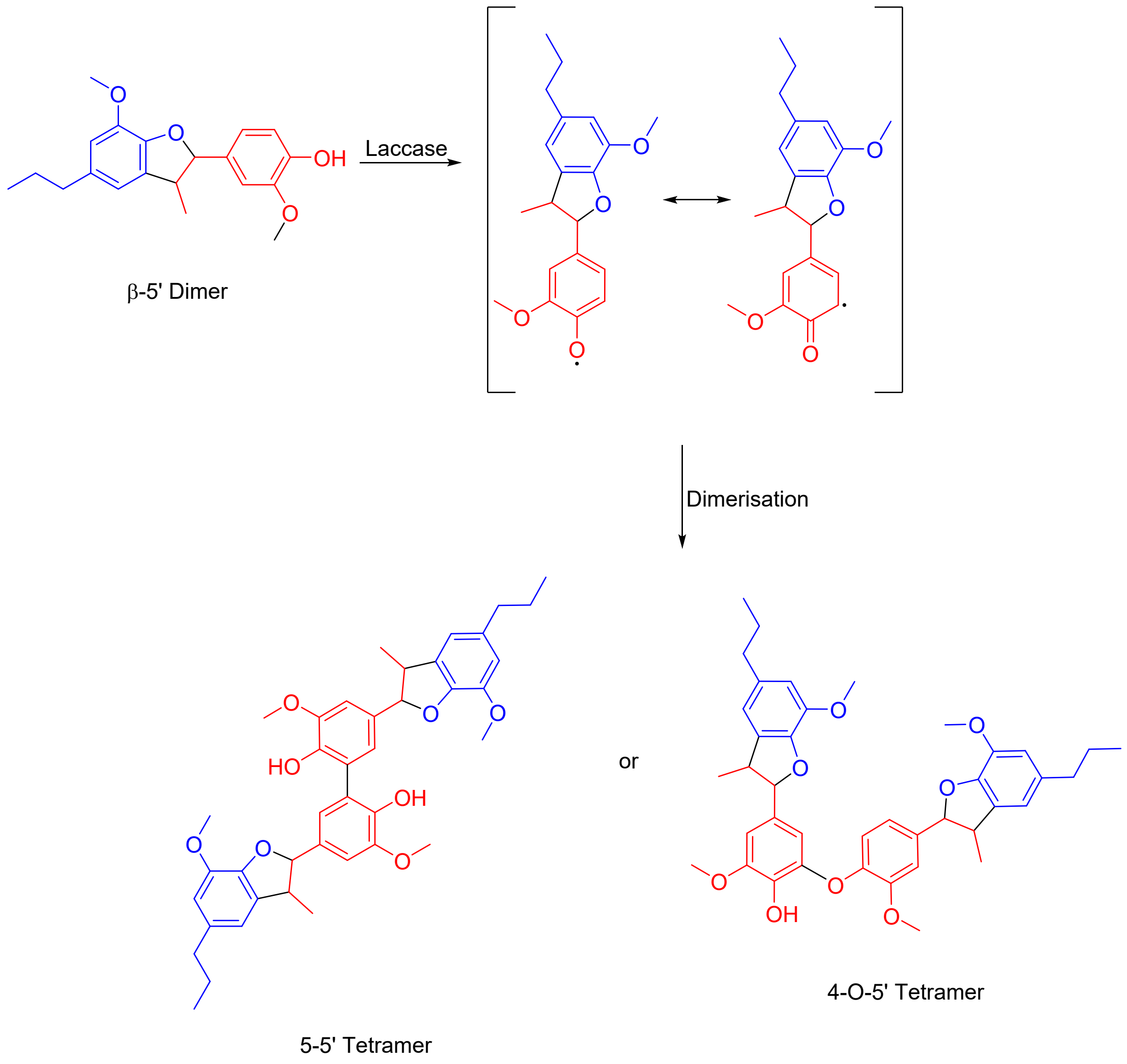


**SI 8: Possible mechanism of dimerisation of the β-5' dimer to form a tetramer**
